# Dauricine Inhibits Macrophages M2 Polarization and Regulates the Progression and Ferroptosis via HCK/IDO1 in Urinary Bladder Cancer

**DOI:** 10.1002/fsn3.71341

**Published:** 2025-12-21

**Authors:** Jie Gong, Dianyu Sun, Yuxin Zheng, Chuwen Mao, Bowen Yu, Xiaogang Li, Yanhua Xuan

**Affiliations:** ^1^ Department of Pathology Yanbian University College of Medicine Yanji China; ^2^ Department of Gastroenterology Yanbian University Hospital Yanji China; ^3^ Department of Urology Jilin City Chemical Industry Hospital Jilin China; ^4^ Department of Urology Yanbian University Hospital Yanji China; ^5^ Key Laboratory of Natural Medicines of the Changbai Mountain, Ministry of Education Yanbian University Yanji China

**Keywords:** bladder cancer, chinese medicine monomer, dauricine, ferroptosis, macrophage

## Abstract

Tumor‐associated macrophages (TAMs), particularly the M2 phenotype, have the ability to promote malignant tumor progression and metastasis. Dauricine (DAU) is a Chinese herbal monomer that has been shown to have inhibitory effects on various tumor cells. However, the mechanisms by which DAU influences tumor microenvironment and bladder cancer (BLCA) progression remain unclear. Herein, we investigated the effects of DAU on macrophage M2 polarization and BLCA progression. In this study, besides demonstrating that DAU can mediate the inhibition of macrophage M2 polarization and promote macrophage ferroptosis induced by BLCA cell supernatants via HCK, we further verified that DAU regulated IDO1, which is downstream of HCK, to affect the M2‐like TAMs induced by BLCA progression and ferroptosis. In addition, we also observed an increase in ferritinophagy in BLCA cells under DAU treatment. These findings reveal an unsuspected function of DAU in inhibiting malignant progression of BLCA by interfering with M2 polarization and ferroptosis in TAMs through the HCK/IDO1 axis.

## Graphical abstract

DAU inhibits macrophage M2 polarization and promotes ferroptosis through HCK. DAU mediates TAMs M2 polarization to inhibit the proliferation, migration, invasion, and in vivo metastasis and reverses the reduction of ferroptosis via the IDO1 pathway in BLCA. DAU impacts the downstream target of HCK/IDO1, which inhibits tumor progression by promoting ferritinophagy.

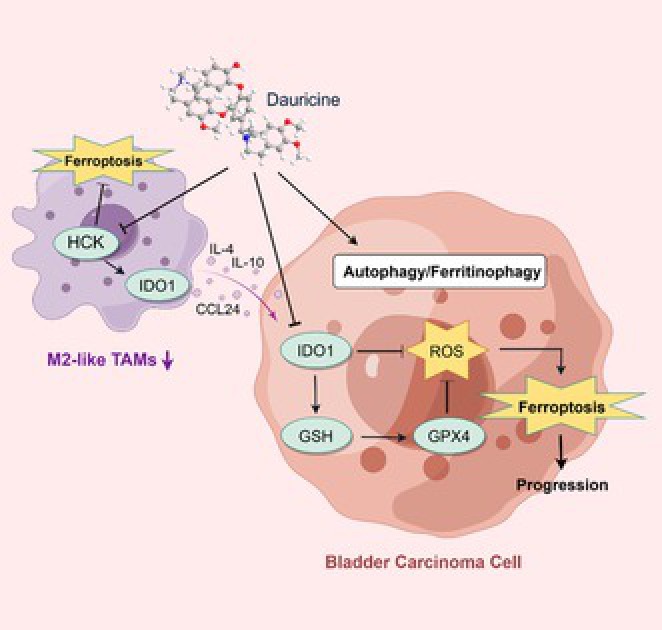

**Abbreviations**
BLCA: bladder cancer
CCK‐8: Cell Counting Kit‐8
CM: Conditioned medium
DAU: Dauricine
GSH: Glutathione
HE: hematoxylin–eosin
IDO1: Indoleamine 2,3‐dioxygenase 1
IF: immunofluorescence
IHC: Immunohistochemistry
iNOS: inducible nitric oxide synthase
NC: negative control
qRT‐PCR: quantitative real‐time PCR
TAMs: Tumor‐associated macrophages
TME: tumor microenvironment
TNFα: tumor necrosis factor α
WB: Western blot

## 1 Introduction

Bladder cancer (BLCA) is the 10th most frequently diagnosed malignancy worldwide, and also the 2nd most common malignancy of the urinary tract, affecting millions of people (Compérat et al. 2022). According to clinical statistics, 90% of all BLCA cases are bladder urothelial carcinoma, of which non‐muscle‐invasive BLCA accounts for 75% and muscle‐invasive BLCA accounts for 25% (Antoni et al. 2017). Although surgical resection, along with adjuvant radiotherapy, chemotherapy, and immunotherapy, can mitigate the high recurrence rate and extend patient survival of BLCA, current treatments have certain toxic side effects on the human body. Therefore, enhanced prevention and early screening efforts, in‐depth research into the molecular mechanisms of BLCA development and prognosis, and the exploration of novel therapeutic targets are urgently needed.

Macrophages constitute one of the vital components within the body's immune system. Depending on the activation state and function, macrophages can have both tumor‐suppressive and tumor‐promoting effects and are polarized into M1 and M2 subtypes. Macrophages that infiltrate tumor tissues or accumulate in the tumor microenvironment are defined as tumor‐associated macrophages (TAMs). These TAMs are predominantly skewed toward the M2 phenotype, referred to as M2‐like TAMs, which could accelerate disease advancement and deteriorate patients' prognosis (Derks et al. 2020). M2‐like TAMs, characterized by the expression of specific surface markers, such as CD163 and CD206, and their anti‐inflammatory nature, dominate the tumor microenvironment (TME), participate in tissue repair, fibrosis, and angiogenesis. They promote the growth and spread of tumor cells through the production of cytokines and chemokines (e.g., CCL2, IL‐6, and IL‐17) (Cassetta and Pollard 2020; Tan et al. 2018; Xiang et al. 2021). A range of pro‐tumorigenic cytokines and chemokines secreted by M2‐like TAMs, including IL‐10 and CCL24, further promote a shift in the polarized state of TAMs toward an M2 phenotype, thereby establishing a positive feedback loop that sustains tumor growth and metastasis. Hence, strategies aimed at reducing macrophage recruitment, reprogramming, or directly depleting M2 macrophages are commonly employed for the treatment of solid tumors (Xia et al. 2020). In addition, M2 macrophages can disrupt the extracellular matrix by secreting a variety of matrix metalloproteinases and pro‐metastatic factors to promote tumor cell proliferation, migration, and invasion by facilitating tumor cell traversal across the basement membrane and extracellular matrix (Nowak and Klink 2020). Notably, recent clinical trials targeting TAMs, such as CSF1R inhibitors and anti‐IL‐10 receptor antibodies, have shown promising efficacy in inhibiting tumor progression, but their application is limited by systemic toxicity and incomplete TAM reprogramming (Belderbos et al. 2019; Sun et al. 2024). This underscores the urgent need for novel therapeutic approaches that can precisely modulate TAM phenotypes while minimizing off‐target effects.

Dauricine (DAU) is a Chinese medicine monomer isolated and extracted from the rhizomes of the traditional medicinal plant *Menispermum dauricum DC*., which was first used in anti‐arrhythmic and anti‐inflammatory treatments. With the current development of Chinese medicine, several studies have shown its inhibitory effect on different tumor cells such as colorectal cancer, pancreatic cancer, melanoma, and glioblastoma (Liu, Yang, et al. 2024). For instance, DAU inhibits melanoma cell proliferation and promotes cell death by inhibiting the Src/STAT3 pathway, which has a strong binding affinity with DAU (Beik et al. 2020). It also can attenuate the stem‐like properties associated with the coactivation of Oct4 and Shh, and cause cell cycle arrest and apoptosis in neuroblastoma and glioblastoma by inhibiting the AKT/β‐catenin signaling pathway. This brings about the inhibition of cell proliferation in neuroblastoma and glioblastoma (Deng et al. 2021). Previous studies of our team have shown that DAU inhibits macrophage M2 polarization and suppresses EMT, migration, and invasion of prostate cancer cells (Li et al. 2023). However, it remains unclear how DAU affects the death mode of macrophages.

Ferroptosis represents an iron‐dependent cell death mode that differs from conventional apoptosis, necrosis, and autophagy (Stockwell et al. 2017). It is tightly related to lipid peroxidation and the imbalance of iron metabolism and plays a pivotal part in the pathogenesis of many diseases, especially in the treatment of cancer (Liu, Kang, and Tang 2022). By understanding the mechanism of ferroptosis, it is possible to overcome tumor resistance or synergy with conventional therapies to enhance therapeutic efficacy, such as chemotherapy; for example, the quinazolinyl‐arylurea derivative 7j can bind to active GPX4 to induce ferroptosis (Zeng et al. 2022). Thus, ferroptosis has been a hot spot for its effects on tumor cells. Recent studies in BLCA have highlighted the therapeutic potential of ferroptosis induction, showing that ferroptosis‐related genes (e.g., SLC7A11, GPX4) are closely associated with tumor progression and immune infiltration (Hu et al. 2023; Liu, Luo, et al. 2024). When it comes to macrophages, iron accumulation is accompanied by the release of ROS and the generation of lipid peroxidation via the Fenton Reaction, inducing ferroptosis of macrophages (Yang et al. 2022). During this process, iron loading and ROS production of macrophages lead to an altered polarization phenotype that promotes macrophages to polarize toward M1, reduces basal and IL‐4‐mediated alternative M2 activation, and causes an obvious escalation in the release and expression levels of inflammatory cytokines (Handa et al. 2019; Yang et al. 2022). Emerging evidence suggests that combining ferroptosis inducers with immune checkpoint inhibitors may amplify antitumor immunity by reprogramming TAMs toward an M1‐like state and enhancing cytotoxic T cell activity, offering a dual therapeutic axis for BLCA (Chen et al. 2024). While the relationship between TAMs and ferroptosis in BLCA has not been clearly investigated.

Thus, modulated susceptibility to ferroptosis is important for attacking tumor cells. One such driver that can regulate ferroptosis is ferritinophagy. The promoting effect of ferritinophagy is through releasing iron stored in ferritin to increase the labile iron pool, followed by enhancing the Fenton reaction and ROS (Wu et al. 2023). Also, overexpression of NCOA4, a major mediator of ferritinophagy, can sensitize cells to ferroptosis by increasing iron availability (Jain and Amaravadi 2022). This process holds significant translational potential, as preclinical studies have demonstrated that ferritinophagy activation enhances the efficacy of cisplatin by amplifying iron‐dependent DNA damage in cancer cells (Hao et al. 2025). This suggests that the research on ferritinophagy in cancer may achieve promoting ferroptosis to suppress tumor growth.

Here, apart from aiming to investigate the effects and mechanism by which DAU inhibits macrophage M2 polarization and thus regulates BLCA progression and ferroptosis, we also focused on DAU's effect on TAMs ferroptosis by targeting their M2 polarization in BLCA. Our study bridges a critical gap in understanding how DAU‐mediated ferroptosis and TAM polarization could be utilized for future clinical BLCA therapy.

## 2 Materials and Methods

### 2.1 Cell Culture

Human bladder cancer cell lines T24 (BDBIO, China), 5637, and mouse macrophage RAW264.7 (Haixing Biosciences, China) had been authenticated and tested for *Mycoplasma*. All these cell lines were cultured in complete medium in a humidified 5% CO_2_ atmosphere at 37°C. Experiments were performed using cells in the logarithmic growth phase.

### 2.2 Conditioned Medium (CM) Preparation

The supernatants of BLCA cells cultured for 48 h were extracted, then cultured with DMEM medium (v/v = 1:1) to have RAW264.7 cells incubated for 24 h to obtain TAMs and supernatants. DAU (MedChemExpress, USA) was added after cell attachment as needed. The specific protocol is referenced from Shang et al. (Shang et al. 2019). Supernatants of TAMs were re‐cultured with BLCA cells by McCoy's 5A or RPMI‐1640 medium containing 10% fetal bovine serum and 1% penicillin–streptomycin (v/v = 1:1) for 48 h. All the supernatants were centrifuged at 2000 rpm for 5 min at 4°C before use to ensure the experimental repeatability.

### 2.3 Western Blot

Following experimental treatment, cell proteins were extracted and lysed by using the RIPA Lysis Buffer supplemented with a protease inhibitor cocktail (Beyotime Biotechnology, China) and quantitated by BCA protein assay kits (Thermo). 30 μg of each protein sample was resolved on 8, 10, or 12% SDS‐PAGE gel, subsequently electroblotted on a PVDF membrane that was cut out according to the molecular weight of the target protein. After being blocked in 5% milk powder (skimmed milk powder: TBS = 1: 20) for 2 h at room temperature (RT), the membranes were incubated at 4°C overnight with primary antibodies detailed in Table 1. After discarding the antibodies, the membranes were washed with TBST three times before incubating with specific secondary antibodies for 2 h at RT.

**TABLE 1:** Antibodies used in western blot, immunohistochemistry and immunofluorescence.

Antibody name	Company	Catalog number
CD206	Proteintech	60143‐1‐Ig
CD163	Biorbyt	orb13303
iNOS	Proteintech	18985‐1‐AP
TNF‐α	Proteintech	17590‐1‐AP
FTH1	CST	3998S
GPX4	Proteintech	67763‐1‐Ig
SLC7A11	Proteintech	26864‐1‐AP
E‐Cadherin	Abcam	ab40772
Vimentin	Proteintech	10366‐1‐AP
Snail	Proteintech	13099‐1‐AP
HCK	Proteintech	11600‐1‐AP
IDO1	Proteintech	13268‐1‐AP
NCOA4	Abcam	ab86707
p62	Proteintech	18420‐1‐AP
LC3B	CST	2775
ATG3	Abcam	ab108251
α‐tubulin	Proteintech	11224‐1‐AP
GAPDH	Proteintech	60004‐1‐Ig
Ki67	Proteintech	27309‐1‐AP

### 2.4 RNA Isolation and Quantitative Real‐Time PCR (qRT‐PCR)

Total RNA was extracted from cells by kits (QIAGEN, Germany). Reverse transcription of RNAs was performed with SuperScript VILO MasterMix (Invitrogen, USA) to synthesize cDNA. A 2 × HQ SYBR qPCR Mix (ZF501) (ZOMANBIO, China) was used for qRT‐PCR. The relative expression levels were determined using the 2^−ΔΔCq^ method, with normalization to GAPDH as the reference gene. Experiments were carried out in triplicate. The primers used in these assays are as Table 2.

**TABLE 2:** Primer sequences used in qRT‐PCR.

Gene name	Primer sequence (5′ → 3′)
CD206	Forward: ctg cag atg gaa aca tct aa
	Reverse: gat cca gat aaa cac atg ct
CD163	Forward: gct gtg gta act tgc atc ctg
	Reverse: gca gta gtg ttc cac cca tca
iNOS	Forward: caa gac aca ctt cac cat aa
	Reverse: att cga tag ctt gag gta ga
TNF‐α	Forward: agt tgt cta aac aat gct ga
	Reverse: aaa ctt tat ttc tcg cca ct
IL‐4	Forward: cca act gct tcc ccc tct g
	Reverse: tct gtt acg gtc aac tcg gtg
IL‐6	Forward: act cac ctc ttc aga acg aat tg
	Reverse: cca tct ttg gaa ggt tca ggt tg
IL‐10	Forward: gac ttt aag ggt tac ctg ggt tg
	Reverse: tca cat gcg cct tga tgt ctg
IL‐17	Forward: tcc cac gaa atc cag gat gc
	Reverse: gaa tgt tca ggt tga cca tca c
CCL2	Forward: cag cca gat gca atc aat gcc
	Reverse: tgg aat cct gaa ccc act tct
CCL24	Forward: aca tca tcc cta cgg gct ct
	Reverse: ctt ggg gtc gcc aca gaa c
CXCL9	Forward: cca gta gtg aga aag ggt cgc
	Reverse: agg gct tgg ggc aaa ttg tt
CXCL10	Forward: gtg gca ttc aag gag tac ctc
	Reverse: tga tgg cct tcg att ctg gat t
CXCL11	Forward: gac gct gtc ttt gca tag gc
	Reverse: gga ttt agg cat cgt tgt cct tt
CXCL12	Forward: att ctc aac act cca aac tgt gc
	Reverse: act tta gct tcg ggt caa tgc
HCK	Forward: cat gat ccg ctg ctg gaa gaa c
	Reverse: tgt caa ggc tgc tgc tga tac t
IDO1	Forward: gcc agc ttc gag aaa gag ttg
	Reverse: atc cca gaa cta gac gtg caa
GAPDH	Forward: gga gcg aga tcc ctc caa aat
	Reverse: ggc tgt tgt cat act tct cat gg

### 2.5 Xenografted Tumor Model

Female BALB/c nude mice (4–6 weeks, GemPharmatech, Jiangsu, China) were used and housed in an SPF animal room. A total of 12 mice were randomly assigned to 4 experimental groups (*n* = 3/group) according to body weight. For the control and DAU alone groups, a subcutaneous injection of 3 × 10^6^ T24 cells (100 μL) was administered into the flank. For the TAMs and TAMs with DAU treatment groups, each mouse was injected with 3 × 10^6^ cells (T24 and RAW264.7 at 2:1, 100 μL) in total into their flanks subcutaneously. Starting 7 days post‐injection, mice received 100 μL normal saline (NS) or DAU (10 mg/kg dissolved in 2% DMSO, 40% PEG 300, 5% Tween‐80, and 53% NS) intraperitoneally twice a week. For metastasis experiments, 1 × 10^6^ cells (T24 only or T24 mixed with RAW264.7 at 2:1, 50 μL) were injected into the tail veins of the mice. Four weeks later, mice were sacrificed, and tumor volume and weight were measured and calculated: (mm^3^) = (Length×Width^2^)/2. Liver metastatic nodules of mice in metastasis experiments were removed and fixed. All procedures were carried out in compliance with the guidelines set by the Yanbian University Animal Ethics Committee (Registration number: No. YD20231027007).

### 2.6 Histopathological Assessment

Immunohistochemistry (IHC) staining, hematoxylin–eosin (HE), and immunofluorescence (IF) staining were conducted for histological assessment, following previous protocols (Che et al. 2024). Perls staining was performed by the Prussian Blue Staining Kit (Servicebio, China). Both IHC and IF results were analyzed by Image J. Immunoreactive score (IRS) = SI (staining intensity) × PP (percentage of positive cells). SI was assigned as: 0 = negative; 1 = weak; 2 = moderate; 3 = strong. PP is defined as 0 = 0%–5%; 1 = 6%–25%; 2 = 26%–50%; 3 = 51%–75%; 4 > 75%. The primary antibodies used in these assays are as Table 1.

### 2.7 Gene Transfection

Small interfering RNAs (siRNAs) targeting IDO1 and non‐targeting siRNA as Negative Control (NC) were purchased from Sangon Biotech (China), which have already been selected in the previous studies (Liang et al. 2021). BLCA cells were transfected with NC and IDO1‐targeting siRNA (siIDO1‐1 and siIDO1‐2) using Lipofectamine 3000 transfection reagent (Invitrogen, USA), adhering to the manufacturer's protocol. 24 h post‐transfection, cells were harvested and assayed for protein expression and mRNA levels of the target of interest.

### 2.8 Cell Counting Kit‐8 (CCK‐8) Assay

Cells were inoculated in 96‐well plates at a density of 1 × 10^4^/well, with three sub‐wells in each group. After treating cells according to the experimental groups, Cell Counting Kit‐8 (APExBIO, USA) solution at the rate of 1:9 to medium was added. After incubation at 37°C for 3 h, the absorbance at 450 nm of the cells was detected by an enzyme labeling instrument.

### 2.9 Colony Formation Assay

500 BLCA cells/well were inoculated and cultured in six‐well plates for 7 days. After the appearance of macroscopic colonies, the cells were washed, fixed, and stained with Giemsa (Sigma‐Aldrich, USA).

### 2.10 Wound Healing and Transwell Assays

After BLCA cells were inoculated into 6‐well plates to be cultured to about 70%–80% fusion, the wounds were scratched vertically with the tip of sterile 200 μL pipettes. After 0, 24, and 48 h, images were taken through an inverted microscope with a randomized light field (10 × magnification). As for transwell, Transwell Chambers containing polycarbonate filters (Millipore, USA) were used for migration assays and invasion assays (with matrigel coating). Suspensions with consistent cell counts were inoculated in the upper chambers (medium containing 1% FBS) and bottom chambers (complete medium). After 24 or 48 h, migrated and invaded cells were fixed and stained by HE, then photographed and analyzed. The results of control groups were set as 1.

### 2.11 Lipid Peroxidation Measurement

The total cellular lipid peroxidation was measured using a C11 BODIPY (581/591) probe (MCE, USA). Cells were treated as indicated and then incubated with C11 BODIPY (10 μM) in fresh medium without FBS for 30 min under 5% CO_2_, 37°C. Images of labeled cells were acquired and analyzed by a fluorescence microscope.

### 2.12 Glutathione (GSH) Measurement

Configured standards, added samples and reagents following the instructions of the GSH content assay kit (Solarbio, China). The absorbance at 412 nm of the assay tubes, the standard tubes, and the blank tube were determined and calculated, respectively.

### 2.13 Network Pharmacology and Bioinformatics Analysis

Genes that DAU might play a role in regulating macrophages were screened by the Comparative Toxicogenomics Database (CTD, https://ctdbase.org/) and GEO databases. The correlation between genes was analyzed by the TISIDB online analysis tool (http://cis.hku.hk/TISIDB/index.php) and Gene Expression Profiling Interactive Analysis (GEPIA2, http://cis.hku.hk/TISIDB/index.php). The TCGA database was used to analyze the expression of IDO1 in BLCA tissues and normal tissues through GEPIA2.

### 2.14 Statistical Analysis

We performed statistical analysis by GraphPad Prism 10.0 software; all experiments were each conducted three times, with the results presented as mean ± standard deviation (SD). Comparisons of results between two groups were made using Student's *t*‐test, and comparisons of results between three or more groups utilized one‐way ANOVA. For the sample of 30 patients, normality tests were performed by the Shapiro–Wilk test. Correlations between genes were analyzed using the Pearson chi‐square test. *p* < 0.05 was considered statistically significant.

## 3 Results

### 3.1 DAU Inhibits Macrophage M2 Polarization

**FIGURE 1:**
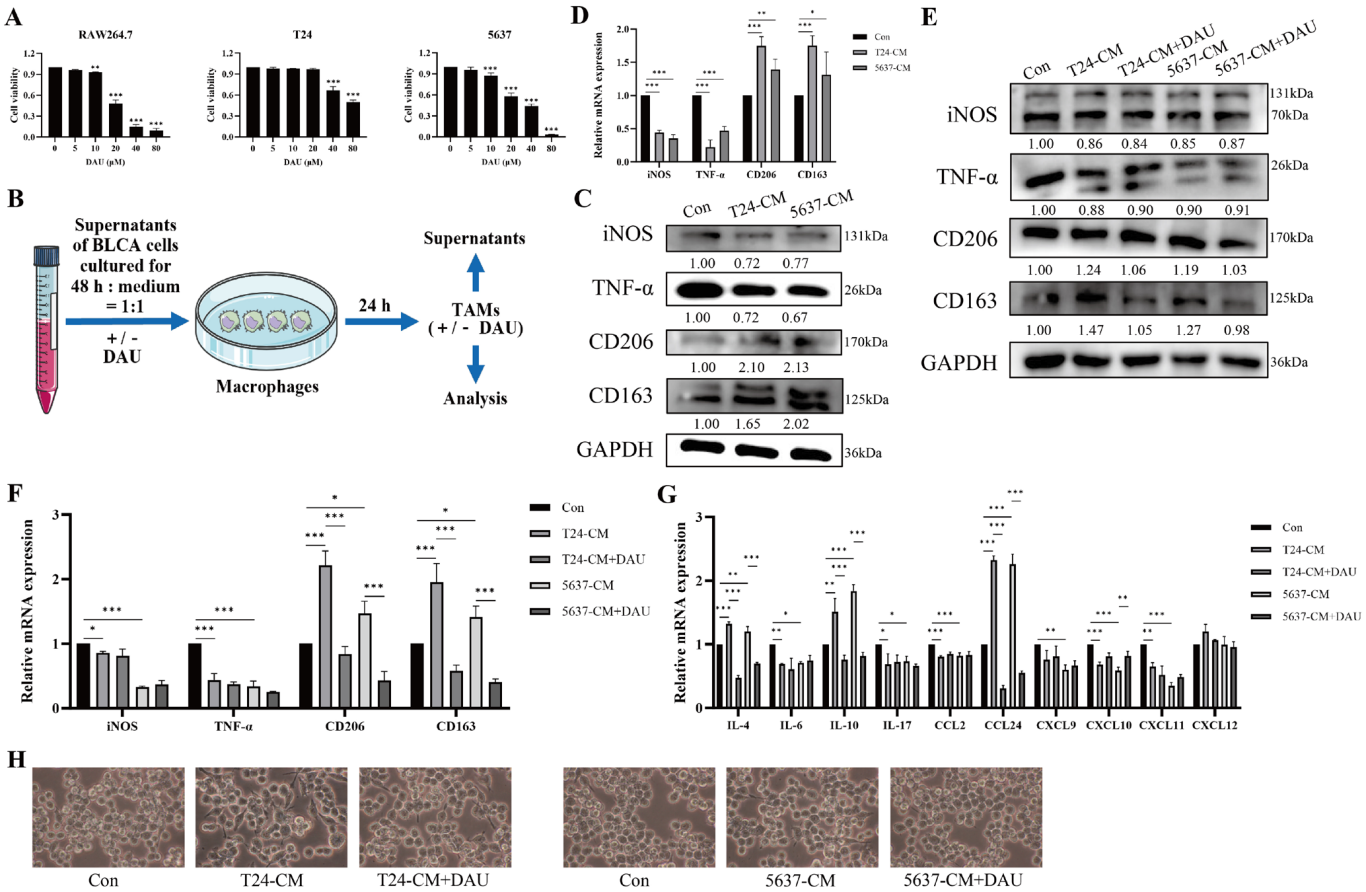
DAU inhibits macrophage M2 polarization. (A) CCK‐8 assay of cell viability of RAW264.7 cells and BLCA cells treated with different concentrations of DAU (0, 5, 10, 20, 40, and 80 μM) for 24 h. (B) Diagrammatic representation of CM system. Western blot (C, E) and qRT‐PCR (D, F) analysis of macrophage M1 and M2 polarization markers in RAW264.7 cells under CM system. (G) Levels of mRNA expression of macrophage‐associated cytokines in RAW264.7 cells. (H) Morphology of RAW264.7 cells (40 × magnification). Data represented mean ± SD (*n* = 3). *p < 0.05; **p < 0.01; ***p < 0.001.

M2 macrophages to facilitate tumor cell proliferation, migration, and invasion by facilitating tumor cell traversal across the basement membrane and extracellular matrix (Nowak and Klink 2020). In this study, we assessed the impact of DAU on the viability of macrophages and BLCA cells. As shown in Figure 1A, DAU exhibited a dose‐dependent inhibitory effect on RAW264.7, T24, and 5637 cells. Since 5 μM DAU showed no cytotoxic effects on these three cell types, it was selected for subsequent experiments.

To verify the effect of DAU on macrophage polarization, a CM system was designed as shown in Figure 1B. We first used supernatants from T24 and 5637 cells to induce macrophage polarization into TAMs. Western blot and qRT‐PCR confirmed that culturing with BLCA cell supernatants decreased M1 markers (iNOS and TNF‐α) and increased M2 markers (CD206 and CD163) expressions (Figure 1C,D). After treatment with DAU, the expressions of M2 markers were restored, while the expressions of M1 markers remained largely unchanged (Figure 1E,F). qRT‐PCR showed that the mRNA expressions of M2‐type cytokines (IL‐4, IL‐10, and CCL24) were decreased in the DAU‐treatment group, but the expressions of M1‐type cytokines (IL‐6, IL‐17, CCL2, CXCL9, CXCL10, and CXCL11) did not show significant changes (Figure 1G). Additionally, cell morphology was observed; M2‐like TAMs showed a slight increase in cell size and morphological growth with pseudopods and protrusions formation, whereas M2‐like TAMs in the DAU‐treatment group showed pseudopods reduction (Figure 1H). These findings suggested that DAU suppressed M2 polarization of macrophages without affecting M1 polarization.

### 3.2 DAU Promotes M2‐Like TAMs Ferroptosis via HCK

**FIGURE 2:**
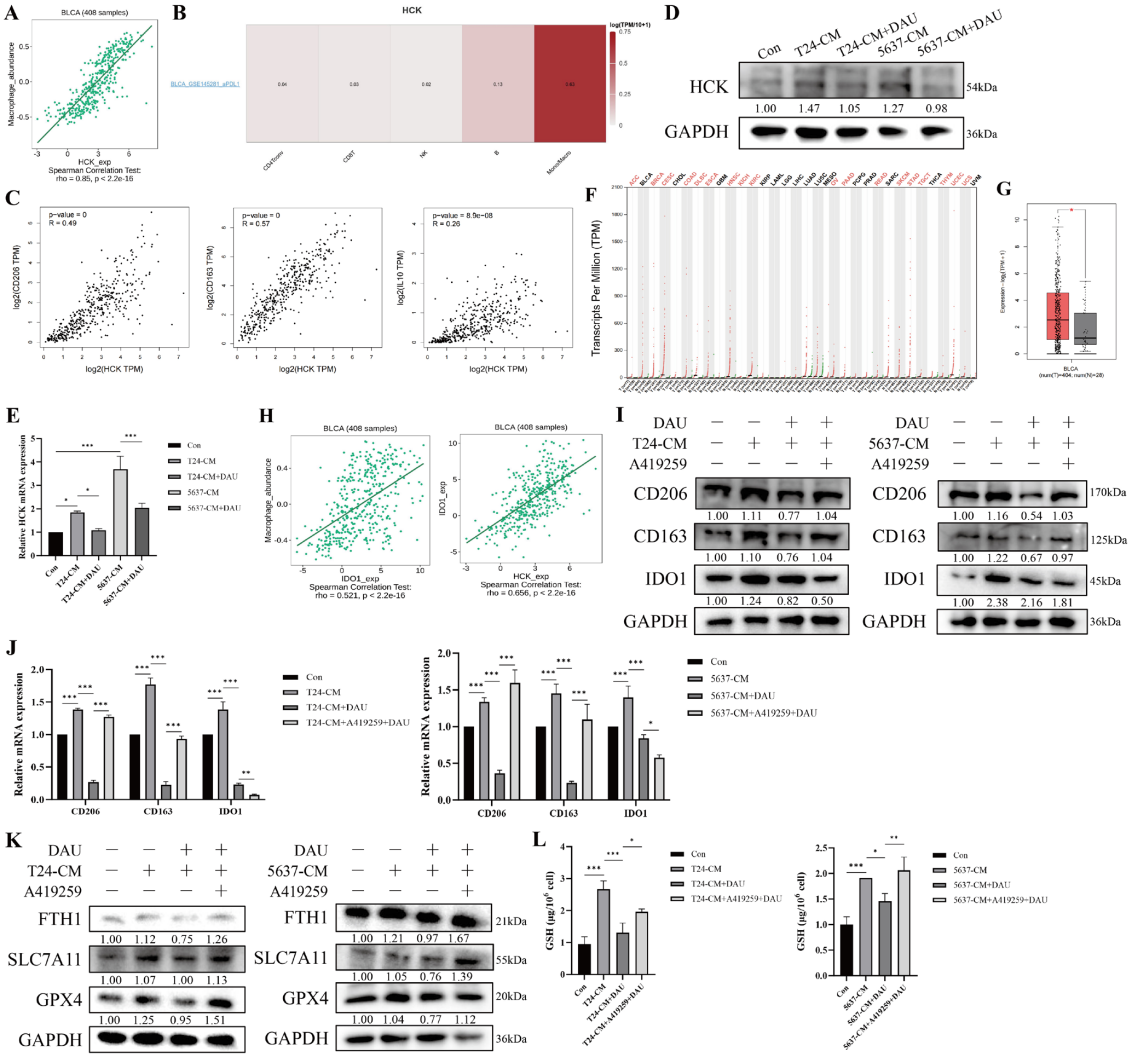
DAU inhibits macrophage M2 polarization and promotes ferroptosis by regulating HCK. (A–C) The correlation between HCK and macrophages was analyzed by TISIDB and GEPIA2 online analysis tool. Western blot (D) and qRT‐PCR (E) analysis of HCK in RAW264.7 cells cultured with BLCA cell supernatants with or without DAU. (F) Dot plot of IDO1 expression profile across all tumor samples and paired normal tissues. Each dot represents the expression of samples. (G) The expression of IDO1 in BLCA tissues and normal tissues was analyzed through GEPIA2. (H) The correlation between HCK and IDO1 in BLCA was analyzed by TISIDB online analysis tool. Western blot (I) and qRT‐PCR (J) analysis of macrophage M2 polarization markers in RAW264.7 cells cultured under the CM system and HCK inhibitor A419259 (1 μM). Western blot (K) and GSH (L) analysis of ferroptosis markers and reduced glutathione in TAMs. Data were represented as mean ± SD (*n* = 3). *p < 0.05; **p < 0.01; ***p < 0.001.

To investigate how DAU affects macrophage M2 polarization, we utilized the CTD and GEO databases to identify genes potentially regulated by DAU in macrophages and identified seven candidate genes. Subsequent validation using the TISIDB and GEPIA2 online tools revealed that, among these genes, only HCK is associated with macrophage phenotype, particularly macrophage M2 polarization, in BLCA (Figure 2A–C). Then, we found that HCK expression levels were significantly increased in M2‐like TAMs, and this phenomenon could be reversed by DAU (Figure 2D,E). Indoleamine 2,3‐dioxygenase 1 (IDO1) is recognized as a novel marker of M2 polarization in macrophages (Najafi et al. 2019). Additionally, IDO1 inhibits tumor ferroptosis through its metabolite kynurenine, which promotes GSH synthesis to enhance cellular resistance to ferroptosis (Fiore et al. 2022). A pan‐cancer analysis using TCGA and GTEx data revealed that IDO1 is highly expressed in BLCA (Figure 2F,G). Results from the TISIDB online tool confirmed a positive correlation between IDO1 expression and both macrophage aggregation and HCK expression in BLCA (Figure 2H). Consequently, we detected M2 markers and IDO1 in M2‐like TAMs treated with the HCK inhibitor (A419259). As illustrated in Figure 2I,J, the expression levels of CD206, CD163, and IDO1 were reduced after DAU treatment and significantly increased upon A419259 treatment. These results demonstrated that DAU‐mediated HCK inhibited M2 polarization of macrophages and IDO1 expression.

Previous studies have demonstrated that ferroptosis can drive TAMs polarization through the release and uptake of proteins (Chen et al. 2021). To investigate the impact of DAU on ferroptosis in M2‐like TAMs, we measured the expressions of ferroptosis‐related proteins and found that the levels of SLC7A11, GPX4, and FTH1 were increased in M2‐like TAMs. Notably, DAU treatment reversed the decrease in ferroptosis observed in M2‐like TAMs. Upon the addition of A419259, ferroptosis was further reduced (Figure 2K). Furthermore, GSH increased in M2‐like TAMs, decreased following DAU treatment, and rebounded after A419259 treatment (Figure 2L). These results suggested that DAU promoted ferroptosis via HCK in M2‐like TAMs.

### 3.3 DAU Inhibits M2‐Like TAMs Induced BLCA Cell Proliferation and Metastasis

**FIGURE 3:**
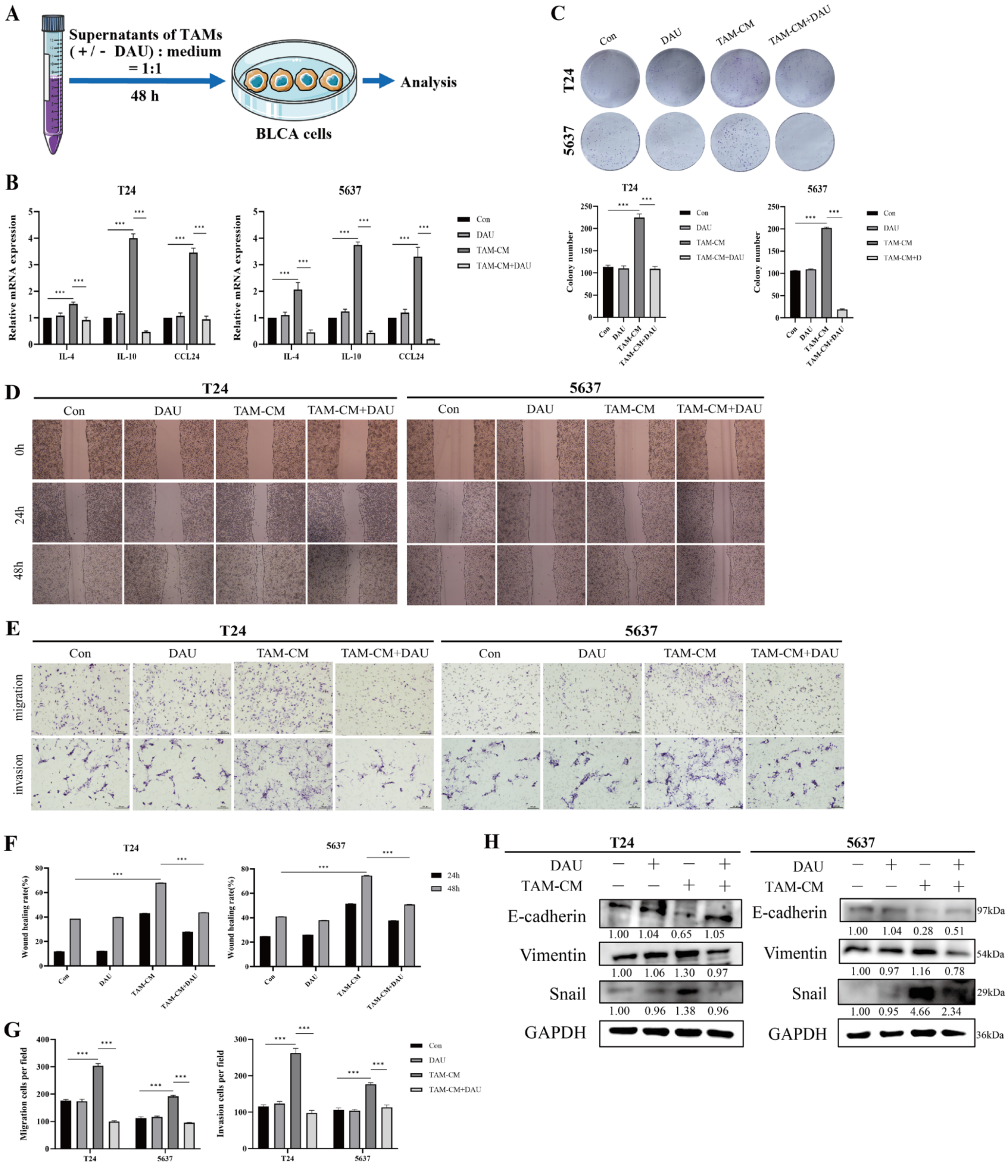
DAU inhibits M2‐like TAMs induced BLCA cell proliferation and metastasis. (A) Illustration of cell processing. (B) mRNA expressions of macrophage‐associated cytokines in BLCA cells cultured with TAMs supernatants for 24 h with or without DAU. Representative images and statistics of colony formation (C), wound healing (D, F), and transwell (E, G) assays, as well as Western blot of EMT markers (H) in TAMs‐induced BLCA cells for 24 h with or without DAU treatment. Data presented as mean ± SD (*n* = 3). *p < 0.05; **p < 0.01; ***p < 0.001.

To investigate the effect of macrophages on tumors after M2 polarization, BLCA cells were cultured with supernatants from M2‐like TAMs, as illustrated in Figure 3A, and the expression of related cytokines and chemokines was assessed. Results showed that cytokines and chemokines associated with M2 polarization were significantly elevated in BLCA cells, and these changes were reversed by DAU treatment (Figure 3B). Further analysis revealed that TAM‐CM significantly enhanced the proliferation, migration, and invasion of T24 and 5637 cells, whereas DAU notably inhibited these effects (Figure 3C–G). Additionally, DAU restored epithelial characteristics by increasing E‐cadherin levels and suppressing the mesenchymal marker Vimentin and the transcription factor Snail, highlighting its ability to mitigate TAMs‐induced EMT progression (Figure 3H).

**FIGURE 4:**
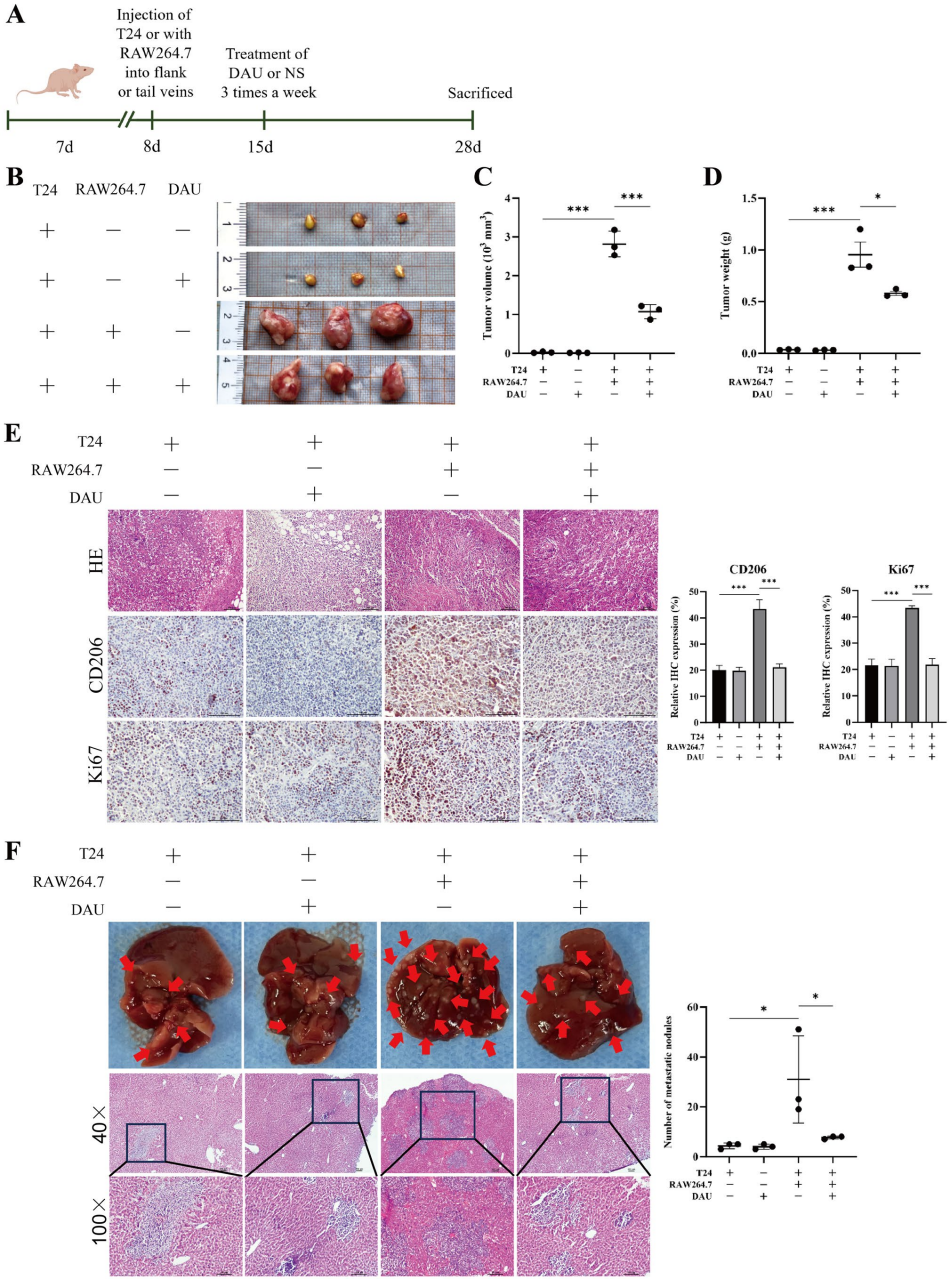
DAU inhibits M2‐like TAMs induced BLCA cell proliferation and metastasis in vivo. (A) Schematic diagram of subcutaneous xenograft model in nude mice. (B) Tumors photo at the end of the experiment. Volume (C) and weight (D) of tumors. (E) Representative images of tumors IHC analysis. (F) Representative images and HE staining of metastatic nodules from nude mice. Data presented as mean ± SD (*n* = 3). *p < 0.05; **p < 0.01; ***p < 0.001.

To further verify the effects of DAU on the proliferation and metastasis of BLCA in vivo, the schematic representation of the experimental workflow is provided in Figure 4A. Co‐injection with BLCA cells and macrophages significantly promoted tumor proliferation and invasion in vivo, which were inhibited after treatment with DAU (Figure 4B–E). DAU treatment reduced liver metastatic nodules, as observed through both visual inspection and HE staining (Figure 4F). Collectively, these results suggest that DAU suppresses tumor proliferation and hepatic metastasis in vivo by decreasing M2 polarization of macrophages in BLCA.

### 3.4 DAU Reverses the BLCA Pro‐Tumorigenic Effects Induced by M2‐Like TAMs Through the Ferroptosis Pathway

**FIGURE 5:**
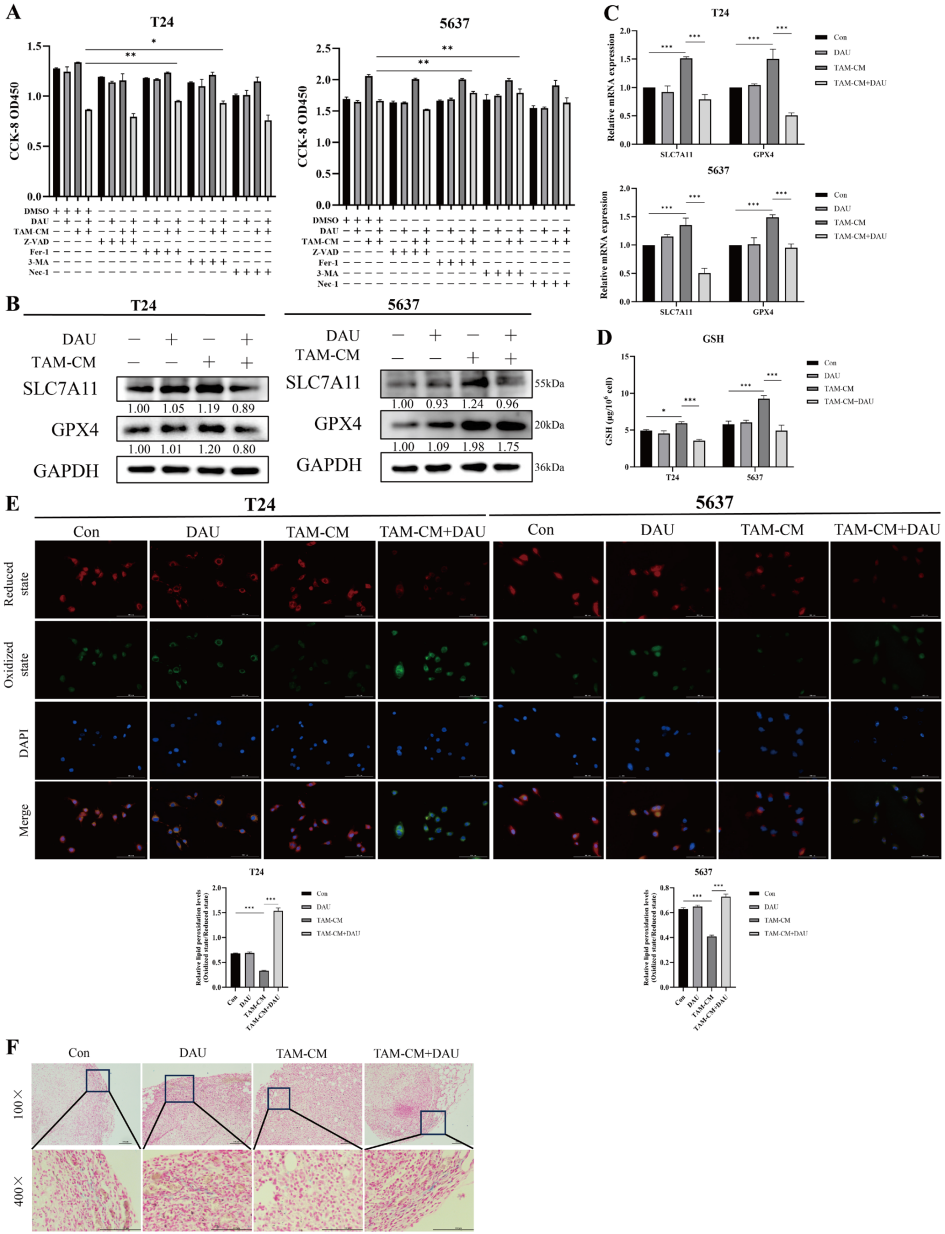
DAU reverses the BLCA pro‐tumorigenic effects induced by M2‐like TAM through the ferroptosis pathway. (A) CCK‐8 assay of cell viability of M2‐like TAMs induced BLCA cells with or without DAU along with various death inhibitors. Western blot (B) and qRT‐PCR (C) analysis of ferroptosis markers in M2‐like TAMs induced BLCA cells and added DAU or not. (D) Reduced Glutathione of BLCA cells by GSH Kit. (E) Lipid peroxidation levels in BLCA cells using C11 BODIPY probe detected by fluorescence microscope (magnification, × 40). (F) Representative images of Perls staining of tumors in nude mice. Data were represented as mean ± SD (*n* = 3). *p < 0.05; **p < 0.01; ***p < 0.001.

To elucidate the mechanism by which DAU‐mediated inhibitions of macrophage M2 polarization suppress the proliferative effect of BLCA cells, various cell death inhibitors were employed, and the proliferative capacity of cancer cells was assessed. As shown in Figure 5A, both the ferroptosis inhibitor (Fer‐1) and autophagy inhibitor (3‐MA) restored cell viability in the DAU treatment group, indicating that DAU inhibited cell survival by promoting ferroptosis and autophagy.

Subsequently, we analyzed the expression of ferroptosis‐related genes in M2‐like TAMs induced by T24 and 5637 cells following the respective treatments. As expected, SLC7A11 and GPX4 levels were elevated in the M2‐like TAM‐CM group, a trend that was rescued by DAU treatment (Figure 5B,C). GSH levels revealed a significant increase in the TAM‐CM group, which decreased after DAU treatment (Figure 5D). Fluorescent image analysis shows that ROS levels were significantly reduced in the M2‐like TAM‐CM group but rebounded after DAU treatment (Figure 5E). Additionally, Perls staining in xenograft mouse tumor tissues revealed reduced Fe^3+^ ion staining in the M2‐like TAM‐CM group, while DAU treatment increased Fe^3+^ ion accumulation (Figure 5F). Collectively, these findings demonstrated that DAU can reverse the BLCA pro‐tumorigenic effects induced by M2‐like TAM through the ferroptosis pathway.

### 3.5 DAU Promotes the BLCA Cell Ferroptosis Induced by M2‐Like TAM‐CM Through IDO1

**FIGURE 6:**
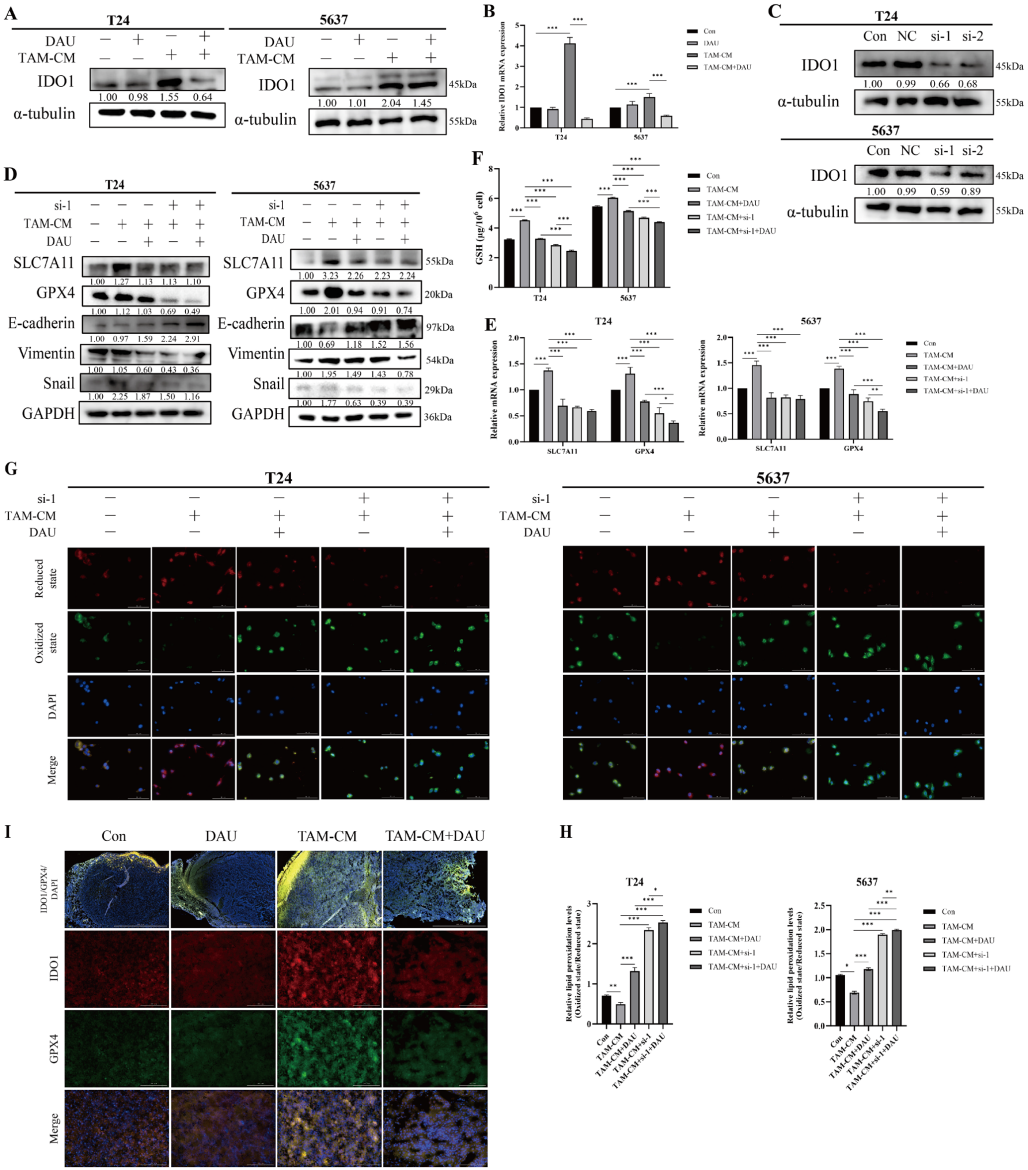
DAU promotes the BLCA cell ferroptosis induced by M2‐like TAM‐CM through IDO1. Western blot (A) and qRT‐PCR (B) analysis of IDO1 in M2‐like TAMs induced BLCA cells treated with DAU or not. (C) The knockdown efficiency of IDO1 in BLCA cells. Western blot (D) and qRT‐PCR (E) analysis of ferroptosis and EMT markers in TAMs‐induced BLCA cells and treated with DAU and IDO1‐siRNA or not. (F) Reduced Glutathione of BLCA cells by GSH Kit. (G, H) C11 BODIPY probe was used to detect lipid peroxidation levels in BLCA cells by fluorescence microscope (magnification, ×40). (I) IF co‐localization of IDO1 and GPX4 in mice tumors. Data were presented as mean ± SD (*n* = 3). *p < 0.05; **p < 0.01; ***p < 0.001.

**FIGURE 7:**
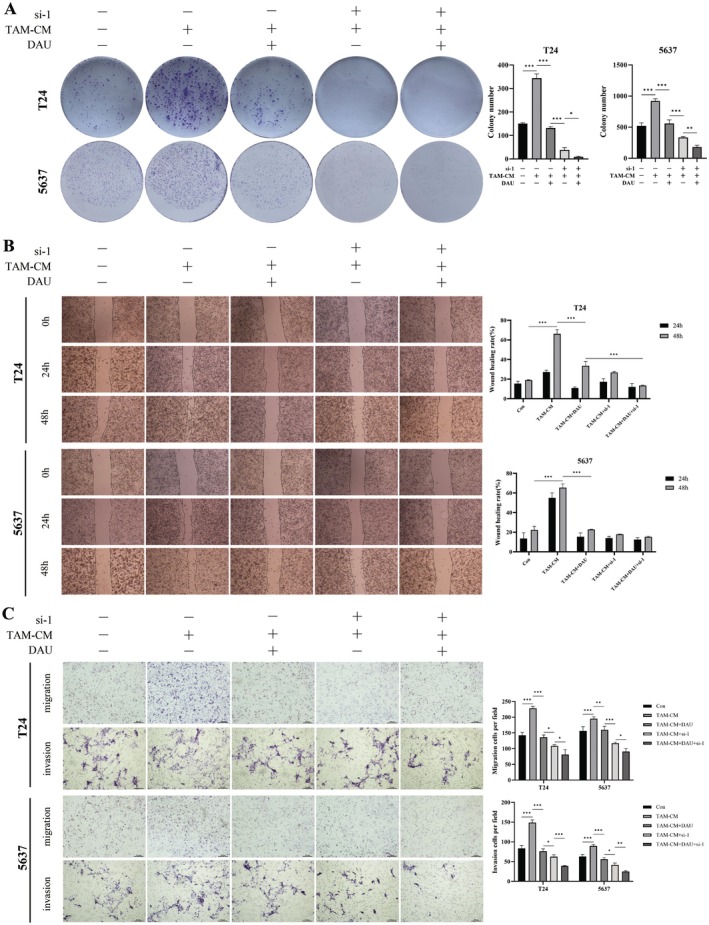
Cell function assays after si‐IDO1 in BLCA cells. Representative images and statistics of colony formation (A), wound healing (B), and transwell (C) assays that were performed in M2‐like TAMs induced BLCA cells with or without DAU treatment and IDO1‐siRNA or not. Data were presented as mean ± SD (*n* = 3). *p < 0.05; **p < 0.01; ***p < 0.001.

Based on the result above that DAU mediates HCK regulation of IDO1 expression, we observed that M2‐like TAM‐CM increased IDO1 expression levels in BLCA cells, an effect that was reversed by DAU treatment (Figure 6A,B). Silencing IDO1 reduced the expressions of SLC7A11 and GPX4, as well as GSH and ROS levels in BLCA cells (Figure 6C). Notably, GPX4 expression was significantly down‐regulated, and the reduction in ferroptosis‐related markers was more pronounced following DAU treatment (Figure 6D–H). To further evaluate the localization relationship between IDO1 and GPX4, we performed IF staining for IDO1/GPX4 in xenograft mice tumor tissues. As shown in Figure 6I, IDO1 and GPX4 expression levels were increased in the co‐injected group, decreased in the DAU treatment group, and IDO1 and GPX4 were partially co‐localized in xenograft tissues. The IHC staining results of 30 tumor tissue samples from BLCA patients provided by Yanbian University Hospital also showed a positive correlation between the expression of CD206, HCK, and IDO1 (Figure S1). Yet, the correlation between the expression of these three genes and clinicopathologic features was not statistically significant (Table S1). Moreover, silencing IDO1 decreased the expression of Vimentin and Snail, along with an upregulation of E‐cadherin expression. The inhibition of EMT was more pronounced following DAU treatment. These findings suggest that DAU reverses M2‐like TAMs induced reduction of ferroptosis and EMT process via IDO1 (Figures 6D and 7A–C).

**FIGURE 8:**
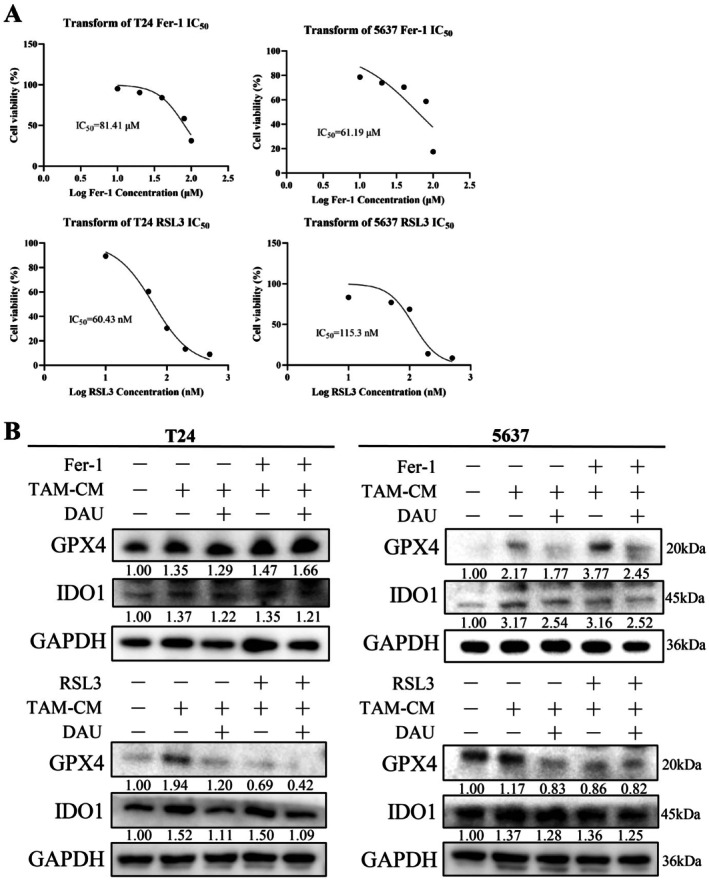
Reversal experiments with the addition of Fer‐1 or RSL3 in BLCA cells. (A) IC_50_ values of Fer‐1 and RSL3 in BLCA cells. (B) Western blot of IDO1 and GPX4 in M2‐like TAMs induced BLCA cells treated with or without DAU with the addition of Fer‐1 or RSL3. Data were presented as mean ± SD (*n* = 3). * *p* < 0.05; ** *p* < 0.01; *** *p* < 0.001.

To explore the contextual relationship between IDO1 and GPX4, we added the ferroptosis inducer (Fer‐1) and the GPX4 inhibitor (RSL3) to M2‐like TAM‐induced cells to observe their reversal effect (Figure 8A). The results showed that Fer‐1 or RSL3 could not affect IDO1 expression in the M2‐like TAM‐CM + DAU group, suggesting that DAU mediated IDO1 to regulate ferroptosis in BLCA cells (Figure 8B).

**FIGURE 9:**
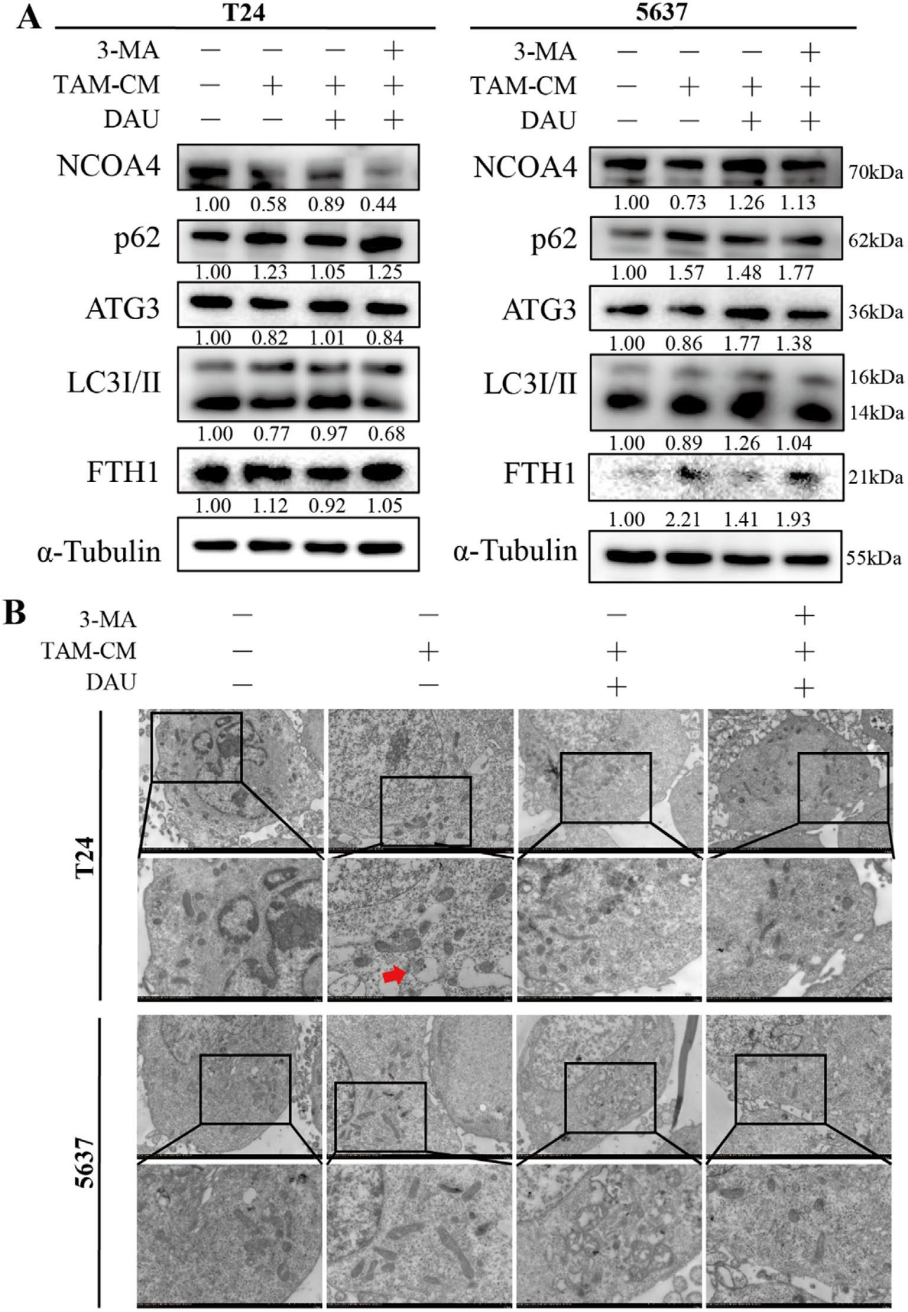
DAU promotes the BLCA cell ferritinophagy. (A) Western blot analysis of ferritinophagy and autophagy markers in TAMs‐induced BLCA cells and treated with or without DAU treatment with the addition of 3‐MA. (B) Images of mitochondria under transmission electron microscopy. Diseased mitochondria are indicated by red arrows. Magnification, ×2.5 (up) and ×5.0 (bottom). Data were presented as mean ± SD (*n* = 3). *p < 0.05; **p < 0.01; ***p < 0.001.

Studies have highlighted that enhanced autophagy promotes ferritin degradation and elevates intracellular iron content, thereby triggering the Fenton reaction and consequent ferroptosis. This process is known as ferritinophagy (Zhang et al. 2021). As previously mentioned, only the ferroptosis inhibitor Fer‐1 and the autophagy inhibitor 3‐MA demonstrated significant effects among the various cell death inhibitors applied (Figure 5A). To investigate whether DAU promotes ferritinophagy, we conducted further analyses by treating M2‐like TAMs induced BLCA cells with the 3‐MA. Western blot analysis revealed that FTH1, a ferritin component, was increased in the M2‐like TAM‐CM group. This increase was reversed by DAU but re‐elevated with 3‐MA treatment. The expression patterns of NCOA4, LC3, and ATG3 were opposite to those of other markers, while the autophagy substrate p62 displayed a similar trend to FTH1 (Figure 9A). Transmission electron microscopy revealed mitochondrial damage following TAM‐CM stimulation, whereas DAU treatment resulted in reduced mitochondrial cristae, with mitochondria appearing morphologically atrophied and swollen (Figure 9B). These findings suggest that DAU reverses M2‐like TAMs induced reduction of ferritinophagy in BLCA cells.

## 4 Discussion

**FIGURE 10:**
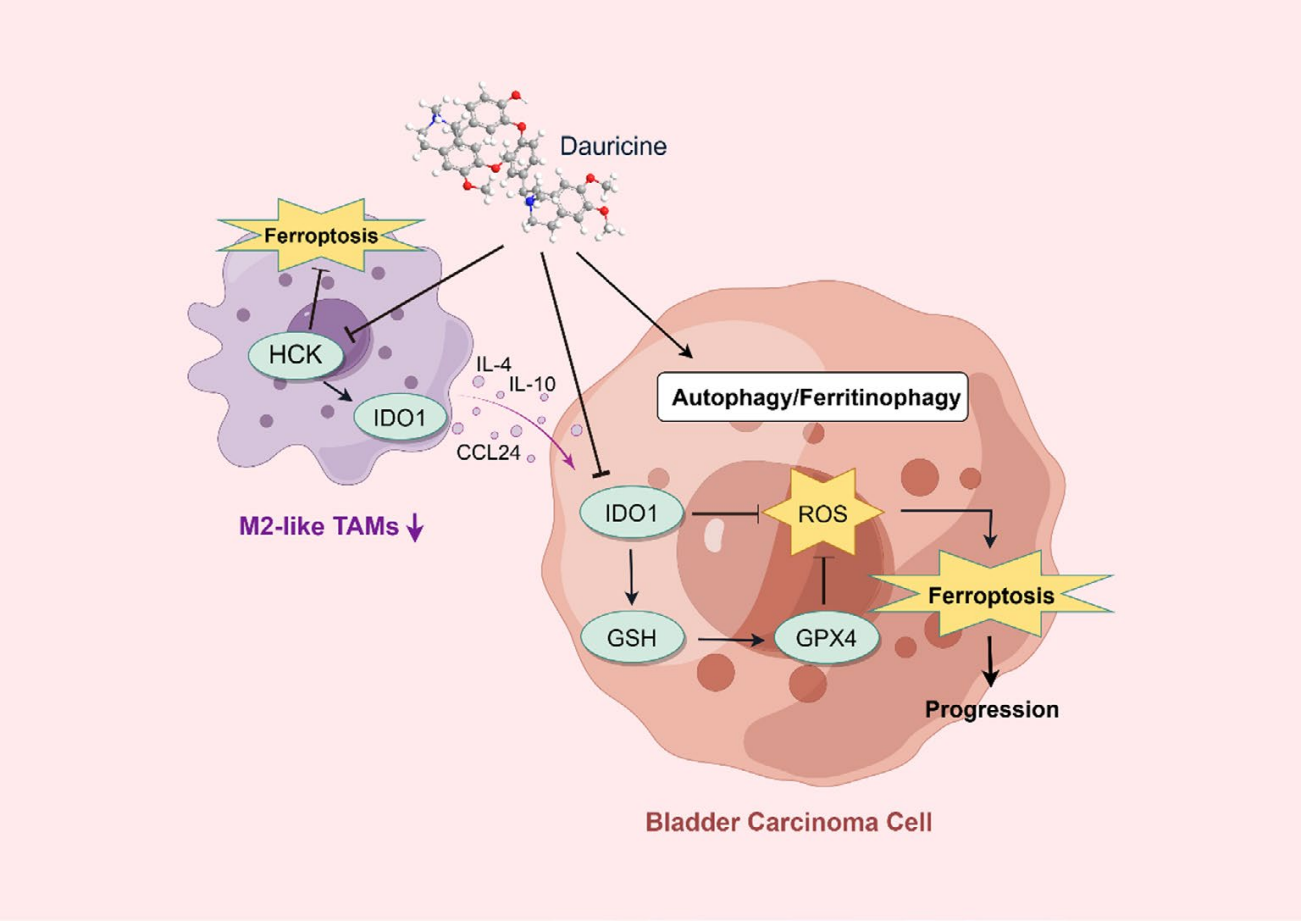
Illustrative model of DAU‐regulated macrophages M2 polarization, and the progression and ferroptosis via HCK/IDO1 in urinary bladder cancer.

DAU is classified as an isoquinoline alkaloid, which demonstrates broad‐spectrum inhibitory effects against various cancer cell types. Further research on DAU, a class of anti‐tumor compounds derived from natural medicinal plants, could facilitate the discovery of therapeutic options with enhanced efficacy and reduced toxicity. In addition, studies have proved that DAU effectively inhibits the proliferation of urinary system tumor cells (Wang et al. 2012). TAMs are multipolarized to an M2 phenotype, and their abundant presence is correlated with increased tumor progression and invasiveness (Jiang et al. 2021; Qian and Pollard 2010). However, the mechanism of interaction between DAU and TAMs in BLCA remains elucidated (Zhang et al. 2019). Our study confirmed the ability of DAU to reverse the inhibitory effect of macrophage M2 polarization and their ferroptosis in the TME, thereby inhibiting BLCA progression and exerting a promotive effect on ferroptosis and ferritinophagy in tumor cells. Consequently, we revealed the specific mechanism of DAU in BLCA treatment and its association with the ferroptosis of both TAMs and tumor cells, which provides an experimental basis and a new way of thinking for the future treatment of BLCA (Figure 10).

Studies have shown that increased TAMs infiltration is strongly linked to malignant progression, lymph node and distant metastasis, reduced survival, and neoangiogenesis in patients with various tumors, including BLCA, lung cancer, invasive breast cancer, cervical cancer, and so on (Braster et al. 2017; Chen, Menon, et al. 2023). M2 macrophages play a critical part in matrix remodeling, tissue repair, and angiogenesis, which consequently thereby promote tumor growth and metastasis. Within the TME, TAMs are progressively polarized toward the M2 phenotype as tumor malignancy progresses. Previous studies of our team have reported that Macelignan efficiently suppresses IL‐4/13‐induced polarization of M2 macrophages by targeting the PI3K/AKT pathway in a ROS‐dependent manner (Che et al. 2024). TAMs can induce apoptosis in renal cell carcinoma cells by modulating the PI3K/AKT signaling pathway, leading to mitochondrial dysfunction and activation of both caspase‐9 and caspase‐3 (Zhang et al. 2018). Our team previously concluded that DAU inhibits macrophage M2 polarization through regulation of the PI3K/AKT signaling pathway and release of CHI3L1 from M2 macrophages, thus mitigating the malignant development of prostate cancer cells induced by M2 macrophages (Li et al. 2023). The role of macrophages in BLCA has been gradually recognized, but the specific mechanisms by which DAU influences macrophage polarization and its downstream effects in BLCA remain currently unclear.

Our findings showed that DAU treatment selectively reduced the expression of M2 polarization hallmarks and associated cytokines in macrophages, while its effect on M1 markers and cytokines was minimal in TAMs. These findings suggest that DAU selectively suppresses the M2 polarization of TAMs induced by BLCA cells, without affecting M1 polarization. Consistent with previous research, our results suggest that modulation of TAMs polarization, particularly inhibition of M2 polarization, is a crucial strategy for mitigating tumor malignant progression. The selective action of DAU on the M2‐like TAMs may provide a more targeted approach to manipulate BLCA TME. The suppression of M2 polarization through the HCK/IDO1 pathway emphasizes the potential of DAU in triggering ferroptosis‐dependent cell death mechanisms in the TME. This strengthens the rationale for considering DAU as an effective agent in inhibiting BLCA progression.

HCK has been implicated in tumor cell survival, with evidence suggesting its overexpression may be allied to tumorigenesis, cancer progression, and poor prognosis (Liu, Shao, et al. 2022; Poh et al. 2022; Wheeler et al. 2009). The overall role of HCK in macrophage activity has remained controversial, with conflicting findings across different cell types and disease conditions. In renal inflammation and fibrosis, HCK inhibition or knockout reduces macrophage M1 polarization and enhances M2 polarization (Xiao et al. 2008). However, in the TME, genetic ablation or chemical inhibition of HCK has shown an opposing effect, reprogramming TAMs and dendritic cells toward a pro‐inflammatory profile (Chen, Wang, et al. 2023). The results of this study align with the latter observation, revealing that BLCA cell supernatants promote macrophage M2 polarization, closely linked to an enhancement of HCK expression in TAMs. This may be due to HCK itself being involved in inhibition or activation of signaling pathways and has a bidirectional regulatory role in signal transduction, which responds differently in different microenvironments. In BLCA TME, HCK may respond to IL‐4, IL‐10, and CCL24 to support M2 polarization. Notably, in this study, DAU significantly inhibited HCK expression in M2 polarization; however, the inhibitory effect of DAU was diminished upon addition of the HCK inhibitor (A419259), which blocks HCK, suggesting that HCK is essential in the DAU‐mediated regulation of macrophage polarization. The biological implication of DAU targeting HCK lies in modulating this kinase's context‐dependent role in immune suppression. In BLCA, inhibition of HCK disrupts its interaction with M2‐skewing markers, such as IDO1, enabling TAMs' M2‐polarized inhibitory and ferroptosis‐prone state. This suggests HCK functions as a molecular switch regulating macrophage plasticity in the TME.

Ferroptosis, a novel form of cell death characterized by iron ion accumulation and ROS, is regulated by various factors including redox homeostasis, mitochondrial activity, lipid and glucose metabolism, and several disease‐related signaling pathways. Macrophage‐derived SLC7A11 significantly reduces ferroptosis activity, promoting TAMs recruitment, infiltration, and M2 polarization, which accelerates hepatocellular carcinoma development and metastasis (Tang et al. 2023). This indicates that inducing ferroptosis in M2 macrophages may act as a pivotal role in tumor suppression. Tang et al. showed that TAMs in hepatocellular carcinoma exhibit M2‐like polarization, SLC7A11 upregulation, and ferroptosis reduction (Tang et al. 2023). Conversely, IL‐6, a marker of macrophage M1 polarization, can promote lipid peroxidation and disrupt iron homeostasis in bronchial epithelial cells, ultimately resulting in ferroptosis (Han et al. 2021; Yang et al. 2022). ROS‐induced p53 acetylation promotes macrophages to polarize to the M1 phenotype via iron accumulation in macrophages (Zhou et al. 2018). These make the repolarization of M2‐like TAMs toward M1 to induce ferroptosis a tumor therapeutic target. To elucidate the mechanisms by which DAU promotes ferroptosis in M2‐like TAMs, we first confirmed that DAU induces ferroptosis in macrophages through inhibition of HCK. These results suggested that DAU not only suppressed M2 polarization but also promoted ferroptosis via HCK in M2‐like TAMs, suggesting that DAU reprograms BLCA TME by both attenuating the pro‐tumorigenic activities of M2‐like TAMs and enhancing ferroptosis‐induced tumor cell death. However, there are limitations to this paper. Future studies should aim to elucidate specific signaling pathways and downstream targets involved in DAU‐mediated HCK regulation and its broader impact on BLCA.

Our study further confirmed that DAU not only suppressed TAMs‐induced BLCA cell proliferation, but also significantly inhibited the migration, invasion, metastasis, and EMT in vitro and in vivo. This suggested that DAU inhibited TAMs‐induced EMT and invasive potential, thereby significantly slowing the progression of BLCA. We further observed that DAU influenced the progression of ferroptosis in BLCA cells. IDO1, a tryptophan‐metabolizing enzyme, is known to be upregulated in many tumor tissues. In the TME, IDO1 not only depletes tryptophan and produces kynurenine to inhibit T‐cell function and promote immune escape, but also influences iron metabolism and ferroptosis sensitivity in tumor cells. Kynurenine can enter cells via the SLC7A11 transporter, promoting cysteine uptake and subsequent glutathione synthesis, which in turn contributes to ferroptosis inhibition (Najafi et al. 2019). This indicates that, beyond its immunosuppressive role, the IDO family enzymes help tumor cells evade ferroptosis‐associated cell death pathways, potentially even promoting tumor progression in a manner independent of immune suppression (Liang et al. 2021; Najafi et al. 2019). Database and bioinformatic analyses revealed a positive correlation between IDO1 expression and macrophage infiltration in BLCA. This research indicated that inhibition of IDO1 expression in BLCA cells reversed M2‐like TAMs‐induced EMT progression and ferroptosis inhibition, which is probably attributed to IDO1's alteration of immune cell composition, such as M2‐like TAMs. This effect was further enhanced by combining DAU treatment with IDO1 inhibition. These findings highlight the potential of DAU in inhibiting BLCA progression and identify IDO1 as a promising therapeutic target, and suggest that targeting ferroptosis pathways may amplify the anti‐tumor effects of IDO1 inhibitors. By simultaneously targeting both HCK and IDO1, DAU intervenes in two pathways, one modulating immune cell phenotype and the other regulating ferroptosis susceptibility, thereby creating a synergistic anti‐tumor effect in BLCA.

Furthermore, ferritinophagy, a selective autophagic degradation process of ferritin, is recognized as a key factor in ferroptosis by increasing intracellular free iron levels. Ferritinophagy mediated by NCOA4 enhances ferroptosis sensitivity in cells by facilitating ferritin degradation and subsequent iron release (Dowdle et al. 2014; Mancias et al. 2014). When intracellular iron is overloaded, macrophages are also capable of triggering ferroptosis through various signaling pathways. Subsequently, using various cell death inhibitors, we confirmed that DAU reduced BLCA cell viability through the induction of ferroptosis and autophagy. Specifically, DAU disrupted cellular iron homeostasis and ROS levels by modulating the expression of SLC7A11 and GPX4, particularly targeting GPX4, a key antioxidant enzyme. The reversal of NCOA4, autophagy‐related proteins, and mitochondrial morphology after the incorporation of 3‐MA also initially demonstrated that DAU could exert its inhibitory effect on BLCA cells through ferritinophagy, illustrating that focusing on ferritinophagy may be an efficient strategy to enhance ferroptosis impaction, especially in tumors such as BLCA, which are ineffective against conventional treatment.

In summary, our study demonstrates that DAU inhibits M2 polarization and induces ferroptosis through the regulation of HCK expression. In BLCA cells, DAU also impacts the downstream target of HCK/IDO1, which inhibits tumor progression by promoting ferroptosis and ferritinophagy. However, this research remains in its early stages, and further preclinical and clinical investigations are essential to validate the efficacy and safety of DAU. Additional investigations should explore the pharmacological properties of DAU, uncover its molecular mechanisms more comprehensively, and assess its potential in combination therapy strategies. This will allow for the optimization of DAU as a candidate for BLCA treatment, assess its potential side effects, and clarify its therapeutic benefits as a candidate for clinical application as an anti‐BLCA agent.

## Author Contributions

**Jie Gong:** conceptualization (lead), data curation (equal), investigation (lead), methodology (equal), supervision (equal), writing – original draft (lead). **Dianyu Sun:** supervision (equal), validation (equal), writing – original draft (supporting). **Yuxin Zheng:** software (lead). **Chuwen Mao:** data curation (equal), methodology (equal). **Bowen Yu:** validation (equal). **Xiaogang Li:** project administration (lead), resources (equal).

## Funding

This study was supported by a grant from the National Natural Science Foundation of China (82160594).

## Ethics Statement

This study was carried out in accordance with the requirements of the Research Ethics Committee of Yanbian University. All experimental procedures adhered strictly to the approved guidelines of the Animal Ethics Committee at Yanbian University. The experimental animals were fed in the Yanbian University SPF animal center (Registration number: SCXK (Jing) 2021‐0011). X.L. and Y.X. are the guarantors of this work and, as such, have full access to all of the data in the study and take responsibility for the integrity of the data and the accuracy of the data analysis.

## Consent

All authors have agreed to publish this manuscript.

## Conflicts of Interest

The authors declare no conflicts of interest.

## Supporting information


**FIGURE S1:** IHC staining of CD206, HCK and IDO1 of BLCA patients' tumor tissues. (A) Representative images of IHC staining. (B) Correlation between the expression of CD206, HCK and IDO1.
**TABLE S1:** The correlation between CD206, HCK and IDO1 expression and clinicopathological characteristics of BLCA patients.

## Data Availability

All data relevant to the study is included in the article or uploaded as the Supporting Information. Other data are available upon request from the corresponding author.
