# Supplementary material for: Dauricine Inhibits Macrophages M2 Polarization and Regulates the Progression and Ferroptosis via HCK/IDO1 in Urinary Bladder Cancer
Source: Food Sci Nutr. 2025 Dec 21;13(12):e71341. doi: 10.1002/fsn3.71341 (PMC12719610; doi:10.1002/fsn3.71341)
Supplement: Supplementary file 1 — FIGURE S1: IHC staining of CD206, HCK and IDO1 of BLCA patients' tumor tissues. (A) Representative images of IHC staining. (B) Correlation between the expression of CD206, HCK and IDO1. TABLE S1: The correlation between CD206, HCK and IDO1 expression and clinicopathological characteristics of BLCA patients. [file FSN3-13-e71341-s001.docx]

**FIGURE S1**


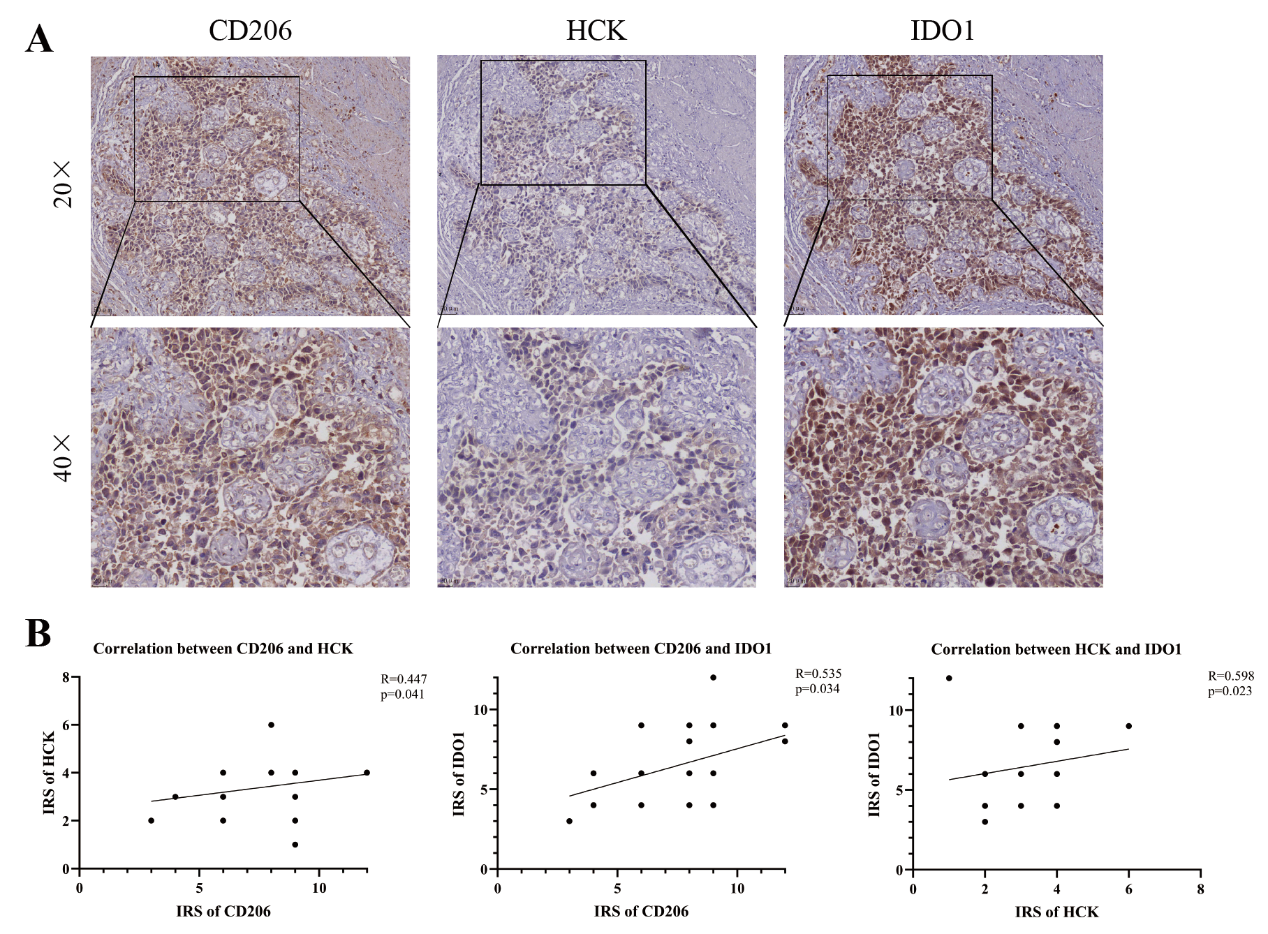


**TABLE S1 |**  The correlation between CD206, HCK and IDO1 expression and clinicopathological characteristics of BLCA patients.

|  | **T stage** | | **N stage** | | **P53** | | **HER2** | |
| --- | --- | --- | --- | --- | --- | --- | --- | --- |
| **Gene** | **R** | ***p*** | **R** | ***p*** | **R** | ***p*** | **R** | ***p*** |
| CD206 | -0.273 | 0.303 | -0.069 | 0.464 | -0.236 | 0.331 | -0.066 | 0.783 |
| HCK | -0.291 | 0.063 | -0.291 | 0.063 | -0.169 | 0.486 | -0.042 | 0.931 |
| IDO1 | -0.408 | 0.010 | -0.134 | 0.464 | -0.133 | 0.582 | -0.201 | 0.611 |
